# Epidemiological and clinical peculiarities of polyglandular syndrome type 3 in pediatric age

**DOI:** 10.1186/s13052-017-0386-4

**Published:** 2017-08-07

**Authors:** Mariella Valenzise, Tommaso Aversa, Angiola Saccomanno, Filippo De Luca, Giuseppina Salzano

**Affiliations:** UOC Pediatria, Department of Human Pathology in Adulthood and Childhood, University of Messina, via Consolare Valeria, 98125 Messina, Italy

**Keywords:** Hashimoto’s thyroiditis, Celiac disease, Type 1 diabetes mellitus

## Abstract

**Background:**

Although the autoimmune polyglandular syndrome type 3 (APS-3) is the commonest APS that may be encountered in pediatric age, last year literature includes only few studies aiming to specifically ascertain the clinical spectrum of APS-3 in childhood and adolescence. Aims of these study were: a) to ascertain how many young patients with apparently isolated Hashimoto’s thyroiditis (HT) are at risk of manifesting other autoimmune disorders (ADs) compatible with APS-3; b) to individuate the ADs which most frequently segregate with HT in the context of an APS-3.

**Methods:**

A selected population of 211 young patients with HT and no risk factors for other APSs was investigated, in order to evaluate the prevalence of other ADs apart from HT and to individuate the ADs which most frequently cluster with HT in the context of APS-3.

**Results:**

The majority of our patients (70.2%) was found to be affected by no other ADs apart from HT, whereas the remaining 29.8% were classified as affected by an APS-3. The ADs which were found most frequently in aggregation with HT were type 1 diabetes mellitus T1DM (61.9%) and celiac disease CD (22.2%).

**Conclusions:**

1) About 30% of young patients with HT may exhibit a clinical picture consistent with APS-3; 2) In the context of APS-3 the ADs that most frequently cluster with HT are T1DM and CD.

## Background

The autoimmune polyglandular sindromes (APSs) are a group of disorders characterized by the combination of multiple autoimmune diseases (ADs). In two of these conditions, i.e. APS-1 and IPEX syndrome, ADs may also be associated with immunodeficiency [1].

According to the original and historical classification of Neufeld et al. [2], APS-3 is a separate clinical entity, that differs from IPEX syndrome for the absence of immunodeficiency and from the remaining APSs for both the mandatory presence of autoimmune thyroid diseases (AITDs) and the mandatory absence of Addison’s disease and hypoparathyroidism [3]. APS-3 is more common than the other APSs and may present even in pediatric age, especially during adolescence and early adulthood [3]. Other ADs which may be observed in the patients with APS-3 are type 1 diabetes mellitus T1DM (APS-3A), celiac disease CD, atrophic gastritis or pernicious anemia (APS-3b), vitiligo or alopecia (APS-3c) and rheumatologic diseases (APS-3d).

Although APS-3 is the most common polyglandular syndrome that may be encountered in pediatric age [4], nevertheless there are only few studies, in the pediatric literature, aiming to specifically ascertain the clinical spectrum of this syndrome in childhood and adolescence [4].

In the present cross-sectional study we have investigated, for the first time, the clustering of extra-thyroidal ADs in a selected population of 211 children and adolescents with pre-existing Hashimoto’s thyroiditis (HT) and no detectable signs of either Addison’s disease or hypoparathyroidism or immunodeficiency, which would not be compatible, by definition, with diagnosis of APS-3 [3].

Our aims were: a) to ascertain how many young patients with HT are at risk of manifesting other ADs compatible with APS-3; b) to individuate the ADs which most frequently segregate with HT in the context of an APS-3.

## Methods

### Patients

The entire study population consisted of 211 young patients (162 girls; median age 11.2 years, range 2.6–22.1), who were admitted to this study according to the following inclusion criteria: a) age < 25 yrs.; b) well-established diagnosis of HT; c) no clinical and/or biological signs of either immunodeficiency or Addison’s disease or hypoparathyroidism at the time of this study.

### Design

All the recruited patients were cross-sectionally investigated, in order to evaluate the prevalence of concomitant extra-thyroidal ADs and to individuate the ADs which most frequently cluster with HT in the context of an APS-3.

### Methods

Diagnosis of HT was assessed according to the following criteria: 1) hypoechogenic thyroid pattern at ultrasonography (US) consistent with AITDs; 2) positive thyroglobulin and/or thyroid peroxidase autoantibodies (TGAbs and TPOAbs, respectively).

TGAb and TPOAb serum concentrations were measured by chemiluminescent immunometric assays. According to these methods, values above 20 or 30 IU/L, respectively, are defined as positive [5–9].

Thyroid US examinations for the assessment of echogenicity were always performed by experienced ultrasonographers with high-resolution US machines.

In our screening approach we took into consideration the following extra-thyroidal ADs: T1DM, CD, pernicious anemia, atrophic gastritis, vitiligo, alopecia areata and idiopathic arthritis. All these diseases were screened by both specific anamnesis and clinical evaluation. Furthermore, all the recruited patients underwent the following autoantibody (Ab) assays: insulin, glutamic acid decarboxylase and islet cell Abs, anti-transglutaminase, anti-gliadin and anti-endomysium Ig G/A, anti-gastric parietal cell Abs and anti-nuclear and anti-smooth muscle Abs. Diagnosis of CD in the patients with specific Ab positivity was confirmed by small intestine biopsy. Diagnosis of both vitiligo and alopecia areata was only based on clinical evaluation [10, 11].

Ab assays were centralized in the same Laboratory of our Department.

The individuals who, at the time of this study, exhibited at least one additional AD, apart from HT, were classified as patients with APS-3 (Group A), whereas those with isolated HT were included in Group B.

The study design was approved by the Ethical Committee of our University Hospital.

### Statistical analysis

It was performed by using SPSS 16.0 version (SPSS, Inc., Chicago, IL, USA).

Frequency rates of the patients with either associated ADs or isolated HT were compared by chi-square test.

Median ages of the patients belonging to Groups A and B were compared between them by Mann–Whitney test.

## Results

In the entire study population the majority of our patients with HT (70.2%) was not found to be affected by any associated ADs, whilst 28.9% were found to be affected by only one extrathyroidal AD and 0.9% were detected to be affected by at least two extra-thyroidal ADs.

On the basis of the established diagnostic criteria 29.8% of the subjects were labelled as patients with APS-3 (Group A), whereas the remaining 70.2% were classified as patients with isolated HT (Group B).

Median ages of the patients belonging to Groups A and B were very similar: respectively 12.2, range 2.6–22.1 yrs. vs 11.0, range 4.1–18.0. In both groups female gender was equally preponderant: respectively 66.7% vs 66.9%.

Among the different extra-thyroidal ADs, the one that was detected most commonly in our study population was T1DM (61.9%), followed by CD (22.2%), vitiligo (6.3%), alopecia areata, idiopathic arthritis and atrophic gastritis (1.6% each). Among these ADs, T1DM was the one that presented earliest, followed by vitiligo and atrophic gastritis (Table 1).

**Table 1:** Median ages (and ranges) at the presentation of the commonest extra-thyroidal autoimmune disorders that were found in the group of 63 patients with autoimmune polyglandular syndrome type 3

	Type 1 Diabetes Mellitus	Vitiligo	Atrophic Gastritis	Alopecia Areata	Celiac disease	Idiopathic Arthritis
Median age	9.7	11.2	11.6	12.8	13.8	16.2
Range	1.6–17.1	6.3–17.3	____	_____	1.4–19.0	____

## Discussion

Among the APSs, the one that has been most extensively investigated in the last years is APS-1, i.e. a rare autosomal recessive disorder due to mutations in the AutoImmune Regulator (AIRE) gene [12, 13]. This syndrome is mainly characterized by the classical triad of chronic mucocutaneous syndrome, hypoparathyroidism and Addison’s disease [14, 15]. More recently, however, many other autoimmune manifestations have been reported to be possibly observed in this condition [16–20].

By contrast, the remaining APSs have been only sporadically investigated in the last few years, although their prevalences are probably higher than that of APS-1 [4].

To the best of our knowledge, this is the first study aiming to specifically ascertain the prevalence of APS-3 in a pediatric study population with isolated HT and no risk factors for the remaining APSs. Furthermore, our study design gave us the opportunity of addressing another intriguing issue, i.e. which may be the preferential clustering of extra-thyroidal ADs in the context of APS-3.

According to our results, the probability that young patients with isolated HT and no risk factors for the remaining APSs might exhibit other ADs compatible with APS-3 may be estimated around 29.8%, at least at a median age of 11.2 years. However, it cannot be excluded that such risk might furtherly increase with increasing patient age.

In fact, it should be considered that the prevalence of T1DM in the general pediatric population increases with age, achieving its zenith between 15 and 19 years [21]. Considering that the median age of the individuals included in our study population was 11.2 years, it would be reasonable to postulate that younger children might not have developed T1DM yet, but they might become diabetic in older age. Furthermore, it has to be underlined that the remaining extra-thyroidal disorders which were detected in this series presented even later than T1DM.

On the other hand, the pivotal role played by age in affecting the development of extra-thyroidal ADs has been just recently confirmed [9].

## Conclusions

About 30% of young patients with HT may exhibit a clinical picture consistent with APS-3. In the context of APS-3 the ADs that most frequently cluster with HT are T1DM and CD.

## Abbreviations

ADs: Autoimmune disorders
APS-1: Autoimmune polyglandular syndrome type 1
APS-3: Autoimmune polyglandular syndrome type 3
CD: Celiac disease
HT: Hashimoto’s thyroiditis
IPEX: Immunodysregulation polyendocrinopathy enteropathy X-linked syndrome
TGAbs: Thyroglobulin autoantibodies
TPOAbs: Thyroid peroxidase autoantibodies
US: Ultrasonography
